# The Moderating Role of the *DYX1C1* Gene in the Effect of Home Supervision on Chinese Children’s Reading Achievements: Evidence from the Diathesis–Stress Model

**DOI:** 10.3390/bs13110891

**Published:** 2023-10-27

**Authors:** Yingnan Niu, He Cai, Li Zhang

**Affiliations:** 1 Collaborative Innovation Center of Assessment for Basic Education Quality, Beijing Normal University, No. 19, XinJieKouWai St., HaiDian District, Beijing 100875, China; niuyingnan@mail.bnu.edu.cn (Y.N.); caihe@mail.bnu.edu.cn (H.C.); 2 School of Sociology and Psychology, Central University of Finance and Economics, 39 South College Road, Haidian District, Beijing 100081, China; 3 Faculty of Psychology, Southwest University, Chongqing 400715, China

**Keywords:** *DYX1C1* gene, home supervision, reading achievements, diathesis–stress model

## Abstract

The current study aimed to explore whether susceptible children (with differences in *DYX1C1* (dyslexia susceptibility 1 candidate gene 1) gene) are more likely to be influenced by either supportive or adverse home supervision in their reading achievements. Home supervision, reading achievements, and genotype data were collected from a total of 745 fourth and fifth grade children and their parents in Chongqing, China. The results showed that there was a significant interaction between the *rs11629841* polymorphism of the *DYX1C1* gene and home supervision on children’s reading achievements. A further analysis based on the re-parameterized regression model showed that the interaction best fit a weak diathesis–stress model, which indicated that the home supervision had a stronger predictive effect on children’s reading achievements among children with the susceptible genotype than children with a non-susceptible genotype in a more adverse environment rather than in a supportive environment. These results suggested that children carrying different genotypes may need targeted interventions and that their parents should emphasize home supervision to develop their children’s reading skills.

## 1. Introduction

In the field of reading research, there are two important concepts, namely reading comprehension and reading competence. Reading comprehension refers to the capacity of an individual to comprehend and interpret the intended message of an author through written text in the most objective manner possible [1]. Meanwhile, reading competence involves the ability to understand, use, reflect, and write text. However, there is currently no consensus on the exact relationship between the concept of reading comprehension and reading competence. Jiménez-Pérez pointed out that reading comprehension is a subset of reading competence, suggesting that reading competence encompasses a broader set of skills beyond just understanding written texts [1]. From this perspective, reading competence pertains to an individual’s ability to effectively apply their reading comprehension skills in various social contexts [1] and this ability can help individuals achieve their goals, develop their knowledge and potential, and eventually integrate into society [2]. Therefore, reading competence has been viewed as one of the most important abilities necessary for people to study and work successfully [3]. According to the research of Rogiers et al., reading performance not only has the potential to predict academic success but also plays a significant role in promoting social participation [4]. Numerous studies, including the work of Ding and Homer, have highlighted the importance of reading proficiency in facilitating the learning of various subjects [5]. Furthermore, international assessments such as the Progress in International Reading Literacy Study (PIRLS), the Programme for International Student Assessment (PISA), and the National Assessment of Educational Progress (NAEP) have all conducted evaluations specifically focused on reading competence. In the past decades, reading education has received an increasing amount of attention in school education [6] because acquiring reading skills is thought to be a prerequisite for all other school-related successes [7].

In contemporary China, traditional reading teaching methods, which were centered around the teacher, focused on knowledge acquisition, and relied heavily on tests, have gradually been phased out. Reading education in modern times, under the guidance of enhancing core competencies, prioritizes the establishment of a favorable curriculum implementation environment that fosters active learning, self-directed learning, cooperative communication, analysis, and problem-solving abilities among students [8]. The “Chinese Language Curriculum Standards for Compulsory Education in China (2022 Edition)” clearly states that by the end of primary school, students’ extracurricular reading should not be less than 4 million words [9]. These data reflect the importance that the education department attaches to students’ extracurricular reading. Indeed, Chinese senior students in primary school are encouraged to engage in extensive reading, with a preference for extracurricular reading materials related to the subject matter. Solely relying on schools for enhancing students’ reading skills through both in-class and extracurricular reading may be challenging, and it necessitates the active involvement of families. Therefore, the collaboration between families and schools plays an indispensable role in improving primary school students’ reading competence. 

However, currently in China, the family–school collaboration system in reading instruction for upper-grade primary school students is incomplete, mainly manifested in the following aspects [10]: First, the teaching evaluation is inefficient, and it cannot promote family–school collaboration through evaluation. Second, the school-based family–school collaboration curriculum is lacking, and it is difficult to improve the level of family–school collaboration based on reading using only textbooks and extracurricular reading materials prepared by parents. Third, family–school collaboration lacks comprehensiveness, and students fail to develop comprehensively in reading activities, thus reducing the quality of family–school collaboration in reading instruction. In summary, it can be seen that in the construction of the family–school collaboration system in upper-grade primary school reading instruction, almost all evaluations emphasize the dominant position of schools. In other words, all evaluations focus on the school side in the construction of the family–school collaboration system, while the family side is relatively neglected. Therefore, it becomes very important to explore and strengthen the family factors that influence children’s reading competence. Meanwhile, compared to other subject areas, such as mathematics, reading achievement has been proven to be more strongly influenced by family factors [11,12], such as parental home supervision. Therefore, exploring the relationship between family factors and children’s reading performance is of great practical significance. Some studies showed that home supervision, which is a widely used rearing strategy, especially in China, was significantly correlated with children’s academic achievement [13,14]. However, some G × E (Gene × Environment interaction) research suggested that the relationship between family factors and children’s behavioral outcomes may be affected by some individual characteristics; specifically, the relationship may be moderated by genes [15,16,17,18]. Thus, the current study aimed to explore whether children carrying different genotypes may have different susceptibilities to the effects of parental home supervision. The results will provide effective suggestions for improving children’s reading achievements.

### 1.1. Home Supervision and Children’s Reading Achievements

The school work of Chinese students is usually conducted under external supervision [19] because the Chinese generally value the virtues of filial piety and the importance of education [19,20]. Therefore, home supervision is a widely used rearing strategy in China [13,14]. Home supervision or home monitoring is a set of correlated parenting behaviors involving attention to and tracking of a child’s whereabouts, activities, and adaptations [21]. Indeed, home supervision has been widely proven to be associated with children’s academic achievements [13,14]; however, the results were mixed. Specifically, on the one hand, some studies have found that home supervision could positively predict children’s academic achievements. A meta-analysis showed that home supervision had a significant but weak relationship with children’s academic achievements compared to other dimensions of parental involvement [22]. Similarly, a longitudinal study analyzed 763 parents and children from the fifth to eighth grade and it showed that, when controlling for demographics, there was a significant positive effect of parental monitoring on GPA (Grade Point Average) [23]. In addition to general home supervision, a meta-analysis was used to examine the relationship between homework checking (one specific home supervision activity) and academic achievement and revealed that it was positively associated with students’ learning [24]. 

However, on the other hand, there were also some inconsistent findings. In some research, a negative relationship was obtained. For example, Guo et al. explored the relationship between family SES (family socioeconomic status) and reading achievement, and the results showed that home monitoring played a critical mediating role between family SES and reading achievement; that is, home monitoring could negatively predict children’s reading achievement, but this effect was only true for girls [25]. Similarly, McNeal’s study revealed that home monitoring was negatively correlated with children’s science achievements and that this effect was moderated by family SES [26]. However, there have been other different findings. Graves et al. found that parental control of children’s TV time may have both positive and negative effects on children’s reading achievements [27]. While Jeynes’s meta-analysis revealed that, although it was positively related to student academic achievements, enforcing household rules did not have significant impacts [28]. Similar to Jeynes’s meta-analysis, the meta-analysis of Tan et al. found that there was no significant relationship between parental supervision of their children and children’s academic achievements (M = 0.01, *p* > 0.05) [29]. However, there are significant variations (Q (df) = 697.78(29), I^2^ = 95.84) in this relationship among different populations, suggesting that there may be potential moderators for this relationship. 

Therefore, the previous findings did not show agreement in the relationship between parental home supervision and children’s reading achievements. These inconsistent findings show that different people may have different susceptibilities to the home supervision rearing strategy and thus demonstrate different relationships between home supervision and children’s reading outcomes. Thus, the current study aimed to explore the differences in the relationship between home supervision and children’s reading achievements from the individual susceptibility perspective, such as genetic factors.

### 1.2. The DYX1C1 Gene and Children’s Reading Achievements

In fact, a number of studies have shown that reading skills have a genetic basis. A twin study showed that reading achievements have a genetic basis of approximately 50 percent or more [30]. Another study also found a high degree of familial clustering of dyslexia, with nine loci identified as being associated with dyslexia in general [31]. Among these foci, the *DYX1C1* (dyslexia susceptibility 1 candidate gene 1) gene at the *DYX1C1* locus was the first identified and widely confirmed susceptibility gene to be associated with dyslexia [32]. Taipale et al. found that nucleotide polymorphisms of the *DYX1C1* gene (−3G > A and 1249G > T) were associated with dyslexia by mapping the breakpoint of a translocation precisely on the short arm of chromosome 15 (15q21DYX1) [33]. 

Recently, several *DYX1C1* loci have been shown to be associated with dyslexia, and among these studies, three polymorphisms were proven to be associated with reading achievements, namely, *rs3743205*, *rs11629841*, and *rs8040756* [34,35,36]. For instance, the *rs3743205* polymorphism of the *DYX1C1* gene affects reading and writing, rapid reading, phonological recall, and spelling abilities by affecting the migration of neurons [34]. The *rs11629841* polymorphism was also found to be significantly associated with dyslexia [37] and the protein encoded by the *rs11629841* polymorphism of the *DYX1C1* gene may be involved in neuronal migration and the development of axons, and therefore, abnormal expression of related genes may lead to disordered neuronal migration and development of axons, further affecting cerebral cortex and thalamus function, resulting in phonological awareness and word-reading deficits [38]. Similarly, *rs8040756* was proven to be strongly related with reading competence in the general Australia population [35]. In this study, the *DYX1C1* gene *rs3743205*, *rs11629841*, and *rs8040756* polymorphisms were selected to investigate their moderating effects on the relationship between home supervision and children’s reading achievements.

### 1.3. Studies on the Interaction of Genes and Environment

**Figure 1:**
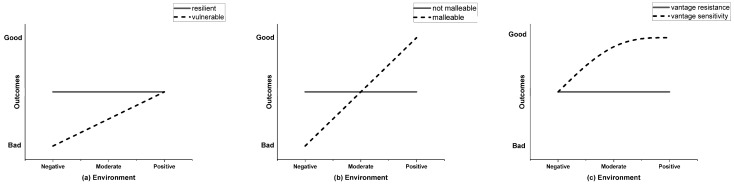
Three genes vs. environment models: (**a**) diathesis–stress model; (**b**) differential susceptibility model; (**c**) vantage sensitivity model. Note: The two lines in each graph represent individuals with different genetic bases. This figure is based on the literatures of Rosenthal [39], Belsky [42], and Sweitzer [44].

Notably, much of the previous research was merely focused on the unilateral effects of genetic or family factors on children’s reading achievements; there were a few studies that have examined the effects of gene and environment interactions on children’s reading achievements. However, the relationship between family factors and children’s reading achievements may be moderated by genes [15,16,17,18]. Currently, there are three mainstream models for the interaction between genes and the environment, namely, the diathesis–stress model, the differential susceptibility model, and the vantage sensitivity model. The diathesis–stress model, originally proposed by Rosenthal [39] to explain the etiology of schizophrenia, uses the term “diathesis” as a synonym for vulnerability, which includes genetic, biological, physiological, cognitive, and personality factors [40]. The diathesis–stress model (see Figure 1a) suggests that individuals who carry risk alleles or susceptible genes are more susceptible to an adverse environment and consequently show problematic behaviors; but, in a supportive environment, whether the individual carries susceptible genes or not, their behavior will not show significant differences [41]. In 1997, Belsky expanded on the diathesis–stress model and proposed the differential susceptibility model based on evolutionary thinking to explain how individuals reproduce [42], that is, the different susceptibility of individuals is a product of evolution, and natural selection mechanisms allow parents to produce offspring with different susceptibilities to the environment, because in uncertain and unpredictable situations, this is the most advantageous way for offspring to reproduce. The differential susceptibility model (see Figure 1b) is based on the idea that individuals carrying susceptible genes will become better in a positive environment and show more behavioral problems in an adverse environment; that is, the individuals carrying susceptible genes will be affected by both favorable and adverse environments [43]. More recently, the vantage sensitivity model has been introduced by Sweitzer [44] to describe the different responses of individuals to positive environmental factors. The vantage sensitivity model (see Figure 1c) assumes that individuals carrying plastic/susceptible genes are more susceptible to positive environments but less susceptible to negative environments, regardless of whether they carry susceptibility genes [45]. All these three models are usually used to explain how different genetic susceptibilities respond to external environmental factors. 

Some studies have supported the diathesis–stress model. For example, one study found that there was an interaction between *DYX1C1*–1259C/G and environmental factors (maternal smoking during pregnancy, birth weight, and family socioeconomic status) and this interaction was consistent with the diathesis–stress model, whereby a less supportive environment combined with a vulnerability gene may be associated with a greater risk of developing overt dyslexia, while the risk may remain relatively constant in a supportive environment [16]. Similarly, another study explored the reading achievement of Han children and found there was a significant interaction between the *rs57809907* polymorphism of the *DYX1C1* gene and the family’s library size which indicated that this interaction was also in line with the diathesis–stress model [18]; that is, when the family’s library size was small, the influence of genetic factors was greater. 

However, in other cases, some studies supported the vantage sensitivity model. The study of Kegel et al. showed that when children with a *DRD4*–gene 7R (7-repeat) allele received positive feedback, they showed higher literacy skills [15]. However, among those who did not receive positive feedback, there were no significant differences in literacy skills among children with different genotypes. Additionally, there were some findings showing that children’s reading performance may be affected by both positive and negative environments, which supports the differential susceptibility model. For instance, Zhao et al. found that there was a significant interaction between CGS of *KIAA0319* and parental education level on reading fluency in Chinese grade three to grade six primary school students and this interaction fit the differential susceptibility model well [46]. Specifically, in a positive environment, characterized by higher parental educational levels, children with a lower CGS of *KIAA0319* demonstrated superior reading fluency compared to children with a higher CGS. Similarly, Plak et al. conducted a randomized controlled trial and found that there was a significant three-way interaction among the CLT pretest, Living Books intervention, and the *DRD4*-gene 7R and that this interaction was consistent with the differential susceptibility model [17]. Specifically, children with delays who carry the *DRD4*-gene 7R benefited the most from the Living Books intervention. 

Therefore, genes have a moderating role on the effect of family factors on children’s reading achievements. In other words, genetic factors result in different susceptibilities and responses to the same environment. Although there have been studies exploring the interaction between family factors such as family socioeconomic status, family’s library size, and parental education level with genetic factors on children’s reading performance, to date, there has been no research investigating how the interaction between home supervision and genetics influences children’s reading performance. As mentioned earlier, home supervision, as a form of parental involvement, is widely used in the Chinese cultural context. However, its relationship with reading performance has yielded inconsistent results, possibly due to moderating factors between home supervision and reading performance, such as variations in different populations. The theoretical models of gene–environment interactions suggest that the relationship between home supervision and reading performance may vary among different susceptibility groups. Therefore, exploring the interaction between genetics and home supervision on children’s reading performance is of great theoretical and practical significance for a better understanding of the underlying mechanisms of home supervision on children’s reading performance. Currently, there may be three potential models for the interaction between genetics and home supervision: (1) the diathesis–stress model, where children carrying susceptibility genes are more susceptible to the negative effects of adverse home supervision, resulting in poorer reading performance, but are unaffected by supportive home supervision, while the reading performance of children without susceptibility genes is not influenced by home supervision; (2) the differential susceptibility model, where children carrying susceptibility genes show improved reading performance under the influence of supportive home supervision, but their reading performance deteriorates under the influence of adverse home supervision, while the reading performance of children without susceptibility genes is not affected by home supervision; and (3) the vantage sensitivity model, where children carrying susceptibility genes show improved reading performance under the influence of supportive home supervision, but their reading performance remains unaffected under adverse home supervision, while the reading performance of children without susceptibility genes is not influenced by home supervision. Therefore, this study aims to explore which model best fits the interaction between home supervision and genetics on children’s reading achievements, namely, how home supervision influences reading achievements among children with different susceptibility levels.

### 1.4. The Current Study

There were two goals for this research. The first goal was to explore the moderating role of the *DYX1C1* gene in the effect of home supervision on Chinese children’s reading achievements. In this study, we used a linear regression model and the RoS (Regions of Significance) method to preliminarily explore the interactions between the *DYX1C1* gene and home supervision on children’s reading achievements. To our knowledge, there are no previous studies that explored the interaction between home supervision and the *DYX1C1* gene in terms of children’s reading achievements and most of the previous studies were based on Caucasian populations. As mentioned above, the effect of family environment on children’s academic achievements may be moderated by genes [15,16,17,18], and an average effect across all participants may mask the effectiveness of home supervision [17]. In addition, as some studies have indicated, the effect of home supervision on children’s reading achievements [47] or the functions played by a particular genotype [48], or even the genes and environment interaction [49,50] may be different among different racial groups or cultures. 

Therefore, the current research first explored the moderating role of the *DYX1C1* gene in the effect of home supervision on Chinese children’s reading achievements, which could explain the relationship between home supervision and children’s reading achievements from a genetic viewpoint. We hypothesized that there was a significant interaction between the *DYX1C1* gene and home supervision on Chinese children’s reading achievements (H1).

The second goal was to model the above interaction. We used a re-parameterized regression model to fully test the three main interaction models. By reviewing previous studies, we found that the previous findings mainly came from testing one or two models rather than directly and fully comparing the three models. In addition, the researchers usually first applied linear regression or ANOVA to test whether there was a significant G × E interaction and then investigated the relationship between the environment and outcome variables in different genotype groups separately or drew an interaction graph to visually observe and determine whether the G × E interaction fit the diathesis–stress model, differential susceptibility model, or vantage sensitivity model [51]. 

However, it is noteworthy that, firstly, the current theoretical models of genes and environment interactions mainly include the diathesis–stress model, differential susceptibility model, and the vantage sensitivity model [50] and the findings from testing one model or two models may weaken the validity of the previous results. Therefore, in this study, the three models were fully tested to determine which model best fits the interaction between *DYX1C1* gene and home supervision on Chinese children’s reading achievements. For each model, the strong and the weak models were distinguished. Specifically, a strong model indicates that an individual who does not carry a susceptibility gene is completely unaffected by the environment while a weak model indicates that the individuals who do not carry a susceptibility gene will also be affected by the environment, but to a lesser extent than the individuals who carry a susceptibility gene. However, there is no difference in the predicted response to the external environment of the individuals who carry the susceptibility gene between the two models [45]. 

Secondly, the traditional exploratory methods mentioned above require multiple tests on the premise that taking into account the main effect of all variables weakens the statistical power of the G × E effect [45] and when the variation explained by the environmental variables is limited, the diathesis–stress pattern or the vantage sensitivity pattern found by traditional methods may actually be part of the differential susceptibility model, which may lead to an incorrect conclusion [50]. 

To examine the above models, we used a re-parameterized regression model to explore the genes and environment interaction [52]; its advantage is that it not only can be used to calculate the value of the crossover point directly but it can also estimate the confidence interval. In contrast to the traditional method, this method can be used directly to judge the properties of the interaction based on the value or position of the crossover point, without a significance test and subsequent analysis of the interaction between the gene and the environment, which can overcome the limitations of exploratory methods [50]. As previous studies have indicated, children’s reading achievements may be affected by either supportive or adverse family factors or both; therefore, we did not make specific hypotheses about which model the interaction in this study might fit best and we did not speculate whether it would be a strong or a weak model.

## 2. Materials and Methods

### 2.1. Participants and Procedure

The participants in the current study were 745 children in fourth grade and fifth grade from three public primary schools in Chongqing, China, and their parents. Specifically, in the 379 students from the fourth grade, there were 181 boys and 198 girls (M_age_ = 9.33 years, SD = 0.52). In the 366 students from the fifth grade, there were 161 boys and 205 girls (M_age_ = 10.25 years, SD = 0.49). The study was approved by the Institutional Review Board of Southwest University. Written informed consent was obtained from students and their parents before the formal tests. Students were required to independently complete the student questionnaires within their classrooms and the DNA extraction work was conducted after the measurement. Trained researchers provided clear instructions to the students. The children were given questionnaires for their parents after school, which included a cover letter explaining the research purpose and process, as well as separate versions for mothers and fathers. The completed questionnaires were collected within a week. The parents independently filled out the questionnaires, which included the home supervision scale as part of the parental involvement questionnaire, as well as information on parental education, occupation, and monthly household income. In special cases, such as illness or business trips, the deadline for returning the questionnaires could be extended to two weeks. 

### 2.2. Measures

#### 2.2.1. Home Supervision

The home supervision in the current study was assessed by the home supervision subscale of the parental involvement questionnaire, which was adapted from Epstein’s “High School and Family Partnership” [53]. The current questionnaire contains 5 items in a single dimension. Each item was rated on a 5-point scale ranging from 1 (never) to 5 (always). The Cronbach’s α for the home supervision dimension was 0.75. The confirmatory factor analysis (CFA) indicated that the model fits well (CFI = 0.971, TLI = 0.942, RMSEA = 0.035). We transformed the original scores to Z scores for further analysis, with higher scores indicating more home supervision.

#### 2.2.2. Reading Achievements

Students’ reading achievements were measured with standardized Chinese tests [54]. The Chinese tests consist of multiple-choice questions and have good reliability and validity [54]. The scores of the Chinese tests were standardized for further analysis, with higher scores reflecting higher reading achievements.

#### 2.2.3. Control Variables

In the current study, the children’s gender (1 = male, 2 = female), age, parental education (1 = primary school or below, 6 = master’s degree or above), parental occupation (1 = national and social management personnel, 10 = urban and rural unemployed or semi-unemployed personnel) and family monthly income (1 = CNY 3000 or below, 5 = CNY 15,001 or more) were used as the control variables in the linear regression model and re-parameterized regression model.

#### 2.2.4. DNA Extraction

DNA was collected from the oral epithelium cells taken from both sides of the mouth using standardized procedures. Genotyping was performed using a Sequenom^®^ MassARRAY^®^ iPLEX Gold (CD Genomics, Shirley, NY, USA) assay [55]. SNPs that passed quality control (QC) criteria (call rate ≥ 95%, minor allele frequency > 0.05, and Hardy-Weinberg disequilibrium *p*-value > 0.01) were retained for genetic analysis. Specifically, *rs3743205* was coded as CT = 0 and TT = 1; *rs8040756* was coded as GG = −1, AG = 0, and AA = 1; and *rs11629841* was coded as GT = 0 and TT = 1. In the final 745 samples, the actual sample size in each gene polymorphism was *rs3743205* CT = 33 (4.43%) and TT = 712 (95.57%); *rs11629841* GT = 155 (20.81%) and TT = 590 (79.19%); and *rs8040756* GG = 539 (72.35%), AG = 189 (25.37%), and AA = 17 (2.28%).

### 2.3. Statistical Analyses

Missing Data: The missing data were handled using the EM imputation method. 

Descriptive Analysis: Pearson correlation analysis was conducted to obtain the correlations among reading achievements, the *DYX1C1* gene, home supervision, and the control variables (age, gender, parental education, parental occupation, and family monthly income). 

Exploratory Analysis: Firstly, we employed a hierarchical regression model to explore whether there was an interaction between the *DYX1C1* gene and home supervision on children’s reading competence. Secondly, we used the RoS (Regions of Significance) method to preliminarily investigate whether the interaction between the *DYX1C1* gene and home supervision have effects on children’s reading performance that align with the diathesis–stress model or the differential susceptibility model. However, since this method can only test the diathesis–stress model and the differential susceptibility model, without considering the vantage sensitivity model, it does not fully test the fit of the three prevailing models to the data. In other words, it only provides preliminary exploration results between the diathesis–stress model and the differential susceptibility model rather than fully comparing the three prevailing gene and environment interaction models. Therefore, it will be only used as an exploratory analysis and we will only consider these results as exploratory results. Specifically, first, the traditional hierarchical regression model was used to examine the interaction between the *DYX1C1* gene and home supervision on children’s reading achievements after controlling for gender, age, parental education, parental occupation, and family monthly income. Second, the RoS (Regions of Significance) method proposed by Roisman et al. was used to calculate the following indicators [39] (http://www.yourpersonality.net/interaction/, accessed on 25 May 2023): (1) Regions of Significance of home supervision (RoS on X): when there are significant differences in the outcome variable (Y) among different genotype subgroups (Z), the value range of the environmental variable (X) is the Region of Significance on X (RoS on X). If there is a significant difference in the outcome variables between different genotype subgroups only when the environmental variable values are low (when dealing with relatively unfavorable environments), it indicates that the G × E conforms to the diathesis–stress hypothesis; if there is a significant difference in the outcome variable between different genotype subgroups when the environmental variable values are both low and high, it indicates that the G × E conforms to the differential susceptibility hypothesis. Moreover, the significance interval of environmental variables is recommended to be within M ± 2 SD. (2) PoI (Proportion of Interaction) and PA (Proportion Affect) indices: the PoI index represents the percentage of “good interaction effect b” to the total interaction effect (the sum of “good interaction effect b” and “poor interaction effect w”), i.e., b/(b + w). Therefore, the closer the PoI index is to 0.50, the more it supports the differential susceptibility hypothesis, and the closer it is to 0, the more it supports the diathesis–stress hypothesis. The PA index represents the percentage of participants affected by good interaction effects, and its value needs to be ≥ 16% to indicate compliance with the differential susceptibility hypothesis. (3) Nonlinear indices (X^2^ and ZX^2^; X refers to the predictor, Z refers to the moderator): if there is a quadratic relationship between the environmental variable (X) and the outcome variable (Y) only in individuals with the “risk allele gene”, there is a high possibility of misinterpreting G × E as fitting the differential susceptibility hypothesis. To check for a non-linear relationship between variables, the significance of X^2^ and ZX^2^ in predicting Y can be tested. If it is significant (indicating a non-linear relationship), further investigation is needed to determine whether the interaction term XZ (i.e., G × E) remains significant after controlling for X^2^ and ZX^2^. (4) When simultaneously testing multiple G × E effects, it is necessary to control for the type I error rate. Therefore, multiple corrections of *p*-values were conducted using the sequential Bonferroni test [56,57]. Based on these indicators, the interaction between the *DYX1C1* gene and home supervision on children’s reading achievements was evaluated to preliminarily determine whether it supports the diathesis–stress or differential susceptibility hypothesis.

Confirmatory Analysis: The interaction between the *DYX1C1* gene and home supervision on children’s reading achievements was examined using the re-parameterized regression model proposed by Widaman et al. [52]. The model is as follows (Equation (1)):

(1) $$Y:\;\;\;\;\;\left\{\begin{array}{c} \;\;\mathrm{GROUP}=1\;\;\;\;\;Y=B_{0}+B_{1}\;\left(X_{1}-C\right)+B_{4}X_{4\;}+B_{5}X_{5}+B_{6}X_{6}+B_{7}X_{7}+B_{8}X_{8}+E\\ \;\;\mathrm{GROUP}=2\;\;\;\;\;Y=B_{0}+B_{3}\;\left(X_{3}-C\right)+B_{4}X_{4\;}+B_{5}X_{5}+B_{6}X_{6}+B_{7}X_{7}+B_{8}X_{8}+E \end{array}\;\;\;\;\;\right.$$

In the model, Y represents the dependent variable, which is the children’s reading achievements. GROUP represents different genotype subgroups. X_1_ represents home supervision. X_4_ to X_8_ are the control variables, including gender, age, parental education, parental occupation, and family monthly income. B_1_ is the regression coefficient of home supervision on children’s reading achievements in the “non-risk/non-plastic genotype” subgroup. B_3_ is the regression coefficient of home supervision on children’s reading achievements in the “risk/plastic genotype” subgroup. B_4_ to B_8_ are the regression coefficients of the control variables (gender, age, parental education, parental occupation, and family monthly income). C represents the crossover point of the slopes of the two genotype subgroups.

If the point estimate and confidence interval of C fall within the range of home supervision values, it indicates a “disordinal” interaction between the *DYX1C1* gene and home supervision, supporting the differential susceptibility hypothesis. Conversely, if the point estimate of C is limited to the maximum or minimum value of home supervision, it indicates an “ordinal” interaction between the *DYX1C1* gene and home supervision, supporting the diathesis–stress hypothesis or the vantage sensitivity hypothesis. In these three models, if B_1_ is constrained to 0 (i.e., the “non-risk/non-plastic genotype” subgroup is not influenced by home supervision), it indicates a strong diathesis–stress/strong differential susceptibility/strong vantage sensitivity model. If this constraint is removed (i.e., the “non-risk/non-plastic genotype” subgroup is also influenced by home supervision but to a lesser extent than the “risk/plastic genotype” subgroup), it indicates a weak diathesis–stress/weak differential susceptibility/weak vantage sensitivity model. Combining the position of the crossover point C and the presence or absence of the restriction of B_1_ = 0, six models can be constructed: the strong diathesis–stress model, weak diathesis–stress model, strong differential susceptibility model, weak differential susceptibility model, strong vantage sensitivity model, and weak vantage sensitivity model. Firstly, the model fit of each model was compared using an *F*-test to determine which model fits the data best. Among these models, the weak differential susceptibility model served as the full model, and all the other models were nested within it. Therefore, all the other models were compared to the weak differential susceptibility model using an *F*-test. If the model fit of any other model was significantly worse than that of the weak differential susceptibility model, the weak differential susceptibility model was accepted and the alternative model was rejected. Otherwise, the weak differential susceptibility model was rejected. Additionally, the AIC (Akaike information criterion) and BIC (Bayesian information criterion) were also used to evaluate the model fit, with lower scores indicating a better fit.

## 3. Results

### 3.1. Descriptive Results

The preliminary analysis showed that home supervision was significantly correlated with the children’s reading achievements (*r* = 0.14), while only the *rs11629841* polymorphism of the *DYX1C1* gene was significantly correlated with the children’s reading achievements (*r* = −0.10). In addition, for control variables, only the children’s age (*r* = −0.39), parental education (*r* = 0.13), and parental occupation (*r* = 0.20) were significantly correlated with the children’s reading achievements (see Table 1).

### 3.2. Exploratory Analysis

In the exploratory analysis, the moderating role of the *DYX1C1* gene was firstly tested after controlling for gender, age, parental education, parental occupation, and family monthly income in the hierarchical regression model. The results showed that only the *rs11629841* polymorphism of the *DYX1C1* gene had a significant moderating effect on the relationship between home supervision and the children’s reading achievements (see Table 2). A simple slope analysis showed that home supervision could only positively predict the reading achievements of GT carriers (*β* = 0.15, *t* = 4.48, *p <* 0.001) but not TT carriers (*β* = 0.07, *t* = 1.95, *p* = 0.05). 

**Figure 2:**
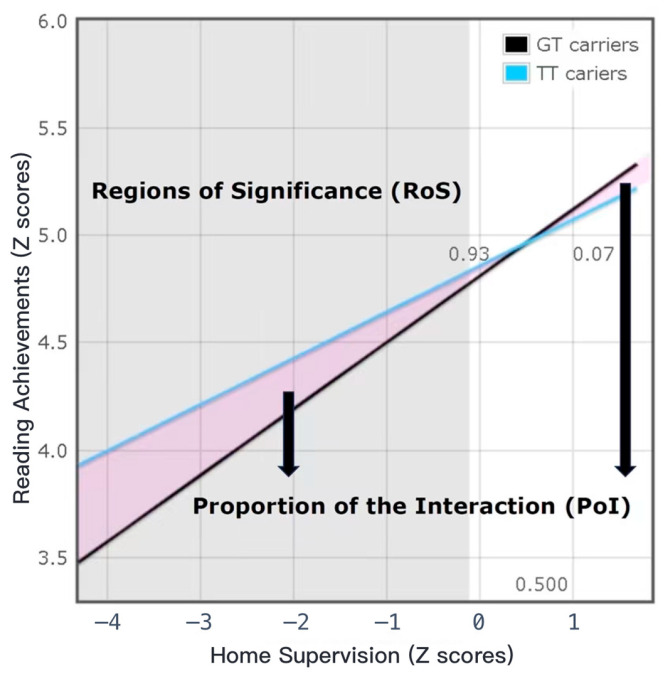
The exploratory results of the interaction between the *rs11629841* polymorphism of the *DYX1C1* gene and home supervision on children’s reading achievements using the RoS method. Note: the purple field in the above figure refers to the proportion of the interaction between the *rs11629841* polymorphism of the *DYX1C1* gene and home supervision on children’s reading achievements. Specifically, 0.07 represents that the proportion of the good interactive effect was 7% (the purple field represented by the black arrow on the right) and 0.93 represents that the proportion of the poor interactive effect was 93% (the purple field represented by the black arrow on the left).

Then, the specific interaction pattern between the *rs11629841* polymorphism of the *DYX1C1* gene and home supervision in the children’s reading achievements was preliminarily tested using the RoS method (see Figure 2). Specifically, (1) the lower and upper boundaries of the Region of Significance for home supervision were −0.11 and 3.83, respectively. This indicates that, for children, when the value of home supervision was below −0.11, GT carriers have significantly lower reading achievements than TT carriers. However, when the value of home supervision was above 3.83, GT carriers have significantly higher reading achievements than TT carriers. However, this significant interval exceeds the range of home supervision values in this study. (2) The PoI index was 0.07, indicating that 7% of the children experienced a good interactive effect, while 93% children experienced a poor interactive effect. The PA index was 0.31, indicating that 31% of the children were influenced by a good interactive effect, while 69% were influenced by a poor interactive effect. (3) The predictive effects of X^2^ and ZX^2^ on the children’s reading achievements were not significant (*p.s.* > 0.05), indicating no nonlinear relationship between the variables. (4) When the sequential Bonferroni test [57] was used to correct for multiple comparisons of *p*-values [58], the above interaction effect remained significant (*p* (0.005) < *p* (i) (0.02)) (*p* (i) refers to the critical value of the significance level after sequential Bonferroni corrections) [57]. In conclusion, all indicators indicate that the interaction between the *rs11629841* polymorphism of the *DYX1C1* gene and home supervision on the children’s reading achievements is consistent with the diathesis–stress hypothesis, where the GT genotype is more sensitive to adverse home supervision.

However, the above exploratory analysis did not take the vantage sensitivity model into consideration; thus, we cannot rule out the possibility of the vantage sensitivity model. Therefore, a confirmatory analysis was conducted to explore which model (diathesis–stress model/differential susceptibility model/vantage sensitivity model) best represents the interaction between the *rs11629841* polymorphism and home supervision on the children’s reading achievements.

### 3.3. Confirmatory Analysis

Considering the exploratory results, we assumed that the GT genotype was a “risk/plastic” genotype, while the TT genotype was a “non-risk/non-plastic” genotype. A re-parameterized regression model [52] was generated to explore the interactive pattern between the *rs11629841* polymorphism and home supervision, where the crossover point C was used to centralize the predictive variable (home supervision).

The model b is the full model, and other models were all nested in the model b. Therefore, in order to determine which model fits the data best, *F*-tests were used to determine whether model b fit better than the other models (a, c, d, e, or f) (see Table 3). Constraining the B_1_ = 0 criterion led to model a. Based on model a and model b, constraining the crossover point C of model a or model b to the highest or the lowest value for home supervision yielded model c (a strong diathesis–stress model) and model e (a strong vantage sensitivity model) or model d (a weak diathesis–stress model) and model f (a weak vantage sensitivity model). In view of this outcome, there is a nested relationship between models a, c, d, e, and f and model b. We directly determined whether the variation (R^2^) explained by models a, c, d, e, and f was significantly decreased compared with that model b when a freely estimated parameter was decreased through a *F*-test. It was found that model a (∆R^2^ = 0.004, *p* < 0.05), model c (∆R^2^ = 0.013, *p* < 0.001), model e (∆R^2^ = 0.028, *p* < 0.001), and model f (∆R^2^ = 0.012, *p* < 0.001) significantly decreased the variance compared to model b, while model d (∆R^2^ = 0.004, *p* > 0.05) did not significantly decrease the variance; therefore, models a, b, c, e, and f were rejected, and model d was accepted. 

**Figure 3:**
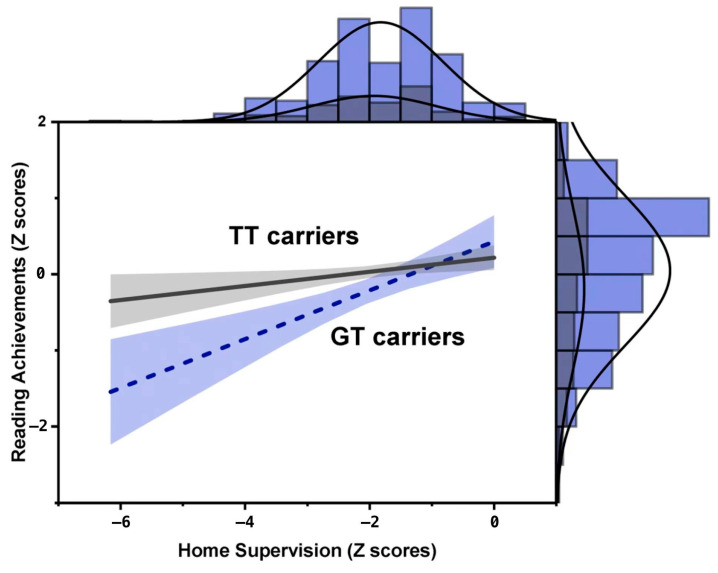
Interaction between the *rs11629841* polymorphism of the *DYX1C1* gene and home supervision on children’s reading achievements in the confirmatory analysis. Note: the purple dotted line represents the relationship between the home supervision and reading achievements in the susceptible children (GT carriers) and the dark grey regular line represents the relationship between the home supervision and reading achievements in the non-susceptible children (TT carriers).

In summary, model d fit the data better than the other models. In other words, the interaction between the *rs11629841* polymorphism of the *DYX1C1* gene and home supervision on children’s reading achievements was consistent with a weak diathesis–stress model (see Figure 3). Specifically, in the GT carriers, parental home supervision could significantly predict the children’s reading achievements (B_1_ = 0.21, SE = 0.07, *p* < 0.001); at the same time, parental home supervision could also significantly predict the children’s reading achievements among TT carriers (B_3_ = 0.10, SE = 0.04, *p* < 0.01), but to a lesser extent.

## 4. Discussion

The current study selected fourth and fifth grade children to participate in the research, using the exploratory method and the confirmatory method [52] to explore the interaction between the *DYX1C1* gene and home supervision on children’s reading achievements and to determine whether this interaction was consistent with the diathesis–stress model, differential susceptibility model, or vantage sensitivity model. Two main conclusions were obtained.

First, the linear regression model revealed that there was an interaction between the *DYX1C1* gene *rs11629841* polymorphism and home supervision on children’s reading achievements; therefore, hypothesis H1 was confirmed. In other words, the *DYX1C1* gene *rs11629841* polymorphism moderated the relationship between home supervision and the children’s reading achievements. Specifically, in GT carriers, home supervision strongly predicted the children’s reading achievements. However, in TT carriers, home supervision had no predictive effect on the children’s reading achievements, which indicates that the G allele may be a “susceptibility gene”. This is consistent with the results of previous studies. For example, by studying the relationship between the *DYX1C1* gene *rs11629841* polymorphism and children’s spelling and orthography skills, Zhang et al. also found that the G allele may be a susceptibility gene [37]; therefore, GT carriers may be more easily affected by the external environment. The RoS method provided preliminary evidence that the above interaction fit the diathesis–stress model well. However, it did not take the vantage sensitivity model into consideration, so an interaction pattern cannot be determined from these results; therefore, a confirmatory method was used to explore which model best fit the interaction.

The re-parameterized regression model showed that the interaction between the *rs11629841* polymorphism and home supervision on the children’s reading achievements fit the weak diathesis–stress model best. That is, GT carriers’ reading achievements were more susceptible to adverse home supervision. TT carriers’ reading achievements were also affected by adverse home supervision, but to a lesser extent. This result revealed that children who carry different alleles might have different responses to home supervision and they only respond to adverse home supervision rather than supportive home supervision. Those who carried the G allele on the *rs11629841* locus of the *DYX1C1* gene were more likely to suffer from less supervision than TT carriers, which means that they need closer parental supervision and control to ensure that they will show good performance on reading, while an indulgent rearing strategy may lead these children into trouble with regard to acquiring reading skills. The reading achievements of the TT carriers were also affected by adverse home supervision but to a lesser extent, which indicated that indulgent parental involvement may rarely lead to poor reading achievements and closer parental supervision might produce weak improvements on their reading performance. The results have important practical implications, especially in the context of Chinese culture, where parents are more likely to adopt stricter rearing strategies for their children. These results suggest that less strict parenting practices may have more negative effects on susceptible children.

This result is consistent with the findings of Mascheretti et al. [16] and seems to be contradictory to the findings of Zhao et al., Plak et al., and Kegel et al. [15,17,46]. The results of the current study found that children who carried the G allele on the *rs11629841* locus of the *DYX1C1* gene were more susceptible to the effects of home supervision than children who carried the T allele. But why are those susceptible children more likely to be influenced by adverse home supervision rather than supportive home supervision? The potential explanations for the diathesis–stress mode in the current study may be related to the Chinese culture. In China, a country that emphasizes filial piety and values education [20], parents generally participate in their children’s academic activities through a supervisory approach [13,14]. Therefore, most students study under external supervision, so children are motivated to learn through external factors [19]. At the same time, education competition in China became intense [59], so parents tend to adopt stricter parenting styles for their children’s study in order not to fail in the educational competition. However, stricter parenting styles do not necessarily lead to significant improvements in academic performance, such as increased reading scores, while relatively indulgent parenting styles can result in children falling behind academically (as children rely more on external motivation for learning, and the lack of external supervision reduces their academic motivation and ultimately lowers their academic performance; other parents strictly control their children, but if you do not exert control, it will lead to lagging behind). Thus, from this perspective, home supervision is more likely to be a necessity rather than for improvement for Chinese children’s reading achievements. Therefore, for susceptible children, they are less likely to benefit from stricter parenting styles, but may experience a decline in reading achievements due to indulgent parental involvement.

## 5. Conclusions

This study reached the following conclusions: (1) The *rs11629841* polymorphism of the *DYX1C1* gene had a moderating role in the effect of home supervision on Chinese children’s reading achievements. The result indicates that the influence of home supervision on children’s reading achievements is indeed different among children with different susceptibilities in grades 4–5 in China’s primary schools. In other words, in terms of improving children’s reading achievements, home supervision, as a strategy in parental involvement, is not applicable to all children. (2) The genes and environment interaction fit the weak diathesis–stress model well. Specifically, children who carried the G allele of the *rs11629841* polymorphism on the *DYX1C1* gene were more susceptible to adverse home supervision than TT carriers, while supportive home supervision may not improve the reading achievements for both categories. In other words, among Chinese primary school children in higher grades, it appears that unfavorable home supervision has a greater impact on the reading performance of susceptible children compared to favorable home supervision. Therefore, when improving the reading performance of susceptible children, more attention should be paid to the unfavorable and negative effects of home supervision in order to reduce the adverse impact on their reading achievements.

These results indicated that it is necessary to apply specific interventions to different children. Parents should not supervise their children equally when improving their reading skills. This intervention is effective for children with the GT genotype, but less effective for those with the TT genotype; therefore, parents should adopt other interventions that are a better fit. These results are of practical importance and advance the research on the genetic mechanisms of children’s reading achievements. In addition, the re-parameterized regression model was used to fully test different genes and environment interaction models, which has the advantage of a robust methodology. In addition, for researchers in didactics or linguistics, it is important to consider the environmental factors (such as home supervision) and genetics simultaneously in studies aimed at improving children’s linguistic competence. Specifically, in terms of family education or the family environment, home supervision plays a significant and positive role in enhancing children’s linguistic competence. However, in teaching practice, it is also crucial to acknowledge the important role genetics plays in the effect of teaching practice on children’s linguistic competence. Children with different susceptibilities may respond differently to the same teaching practices, ultimately demonstrating varying levels of linguistic competence. Therefore, in teaching practice, considering children’s varying susceptibilities to the same environment and identifying susceptible children to implement targeted intervention strategies holds great practical significance in improving children’s linguistic competence.

However, there are some aspects that can be improved with further study. First, the sample size in this study was limited. Future studies need to use a larger sample size to verify the results of this study. In addition, this study only investigated the effect of the interaction between genes and environments on children’s reading achievements. Some studies have shown that the interaction between genes also has a significant effect on the children’s reading achievements [31]. For example, studies by Mascheretti et al. found that *GRIN2B* regulated the correlation between the *DYX1C1* gene [60], *KIAA0319*/*TTRAP*, and short-term memory, which were all found to be related to children’s reading achievements [61]. Future studies could therefore examine the effects of gene–gene interactions on children’s reading achievements. Finally, this study was a cross-sectional study, and longitudinal studies should be conducted to examine the causal relationship between home supervision and children’s reading achievements.

## Figures and Tables

**Table 1:** Descriptive results of *DYX1C1* gene, home supervision, control variables and children’s reading achievements.

Variable (s)	M ± SD	N	1	2	3	4	5	6	7	8	9	10
1. Gender	1.54 ± 0.50	745	1.00									
2. Age	9.78 ± 0.69	745	−0.06	1.00								
3. Z PE	0.00 ± 1.00	745	0.05	−0.04	1.00							
4. Z PO	0.00 ± 1.00	745	−0.11 **	−0.17 **	−0.04	1.00						
5. Z MI	0.00 ± 1.00	745	0.03	−0.02	−0.36 **	−0.06	1.00					
6. Z HS	0.00 ± 1.00	745	0.04	−0.01	0.11 **	0.00	0.11 **	1.00				
7. Z *rs11629841*	0.00 ± 1.00	745	0.01	0.08 *	0.04	−0.09 *	−0.00	−0.04	1.00			
8. Z *rs3743205*	0.00 ± 1.00	745	0.06	0.05	0.05	−0.03	0.03	−0.02	0.08 *	1.00		
9. Z *rs8040756*	0.00 ± 1.00	745	−0.03	−0.03	−0.03	0.11 **	−0.01	0.02	−0.14 **	−0.46 **	1.00	
10. Z RA	0.00 ± 1.00	745	0.06	−0.39 **	0.13 **	0.20 **	0.06	0.14 **	−0.10 **	−0.06	0.04	1.00

Note: M = mean, SD = standard deviation, Z PE = parental education (standardized Z scores), Z PO = parental occupation (standardized Z scores), Z MI = monthly income (standardized Z scores), Z HS = home supervision (standardized Z scores), Z RA = reading achievements (standardized Z scores). * *p* < 0.05, ** *p* < 0.01.

**Table 2:** The hierarchical regression model of the *DYX1C1* gene, home supervision, and their interactions on children’s reading achievements.

Variable(s)	Model 1		Model 2		Model 3	
	β	***t***	β	***t***	β	***t***
Gender	0.04	1.32	0.04	1.28	0.04	1.28
Age	−0.36 ***	−10.59	−0.35 ***	−10.48	−0.35 ***	−10.51
Z Parental education	0.11 **	3.00	0.10 **	2.84	0.11 **	3.02
Z Parental occupation	0.15 ***	4.30	0.14 ***	4.16	0.14 ***	4.13
Z Monthly income	0.03	0.70	0.01	0.40	0.01	0.27
Z Home supervision			0.12 ***	3.71	0.12 ***	3.62
Z *rs11629841*			−0.05	−1.57	−0.05	−1.41
Z *rs3743205*			−0.04	−1.08	−0.04	−1.07
Z *rs8040756*			−0.01	−0.28	−0.01	−0.24
Z Home supervision × Z *rs11629841*					0.09 **	2.85
Z Home supervision × Z *rs3743205*					0.01	0.17
Z Home supervision × Z *rs8040756*					−0.02	−0.60
R^2^	0.19		0.20		0.20	
*F*	33.71 ***		21.14 ***		16.79 ***	
ΔR^2^	0.19		0.02		0.01	
Δ *F*	33.71 ***		4.61 ***		3.18 *	

Note: * *p* < 0.05, ** *p* < 0.01, *** *p* < 0.001.

**Table 3:** Re-parameterized regression model of the interaction between the *rs11629841* polymorphism of the *DYX1C1* gene and home supervision on the children’s reading achievements.

Parameters	Re-Parameterized Regression Model					
	Differential Susceptibility Model		Diathesis–Stress Model		Vantage Sensitivity Model	
	Strong: Model a	Weak: Model b	Strong: Model c	Weak: Model d	Strong: Model e	Weak: Model f
B_0_	4.97 (0.50) ***	5.01 (0.50) ***	4.93 (0.50) ***	5.14 (0.53) ***	4.97 (0.53) ***	4.42 (2.68) ***
C	0.39 (0.28)	0.49 (0.39)	1.85 (–) ^a^	1.85 (–) ^a^	−4.31 (–) ^a^	−4.31 (–) ^a^
95% CI of C	[−0.17, 0.94]	[−0.26, 1.25]	– ^a^	– ^a^	– ^a^	– ^a^
B_1_	0.00 (–) ^a^	0.07 (0.04) *	0.00 (–) ^a^	0.10 (0.04) **	0.00 (–) ^a^	0.13 (0.04) ***
B_3_	0.31 (0.07) ***	0.31 (0.07) ***	0.14 (0.07)	0.21 (0.07) ***	−0.01 (0.07)	0.11 (0.07)
B_4_	0.09 (0.07)	0.08 (0.07)	0.09 (0.07)	0.08 (0.07)	0.09 (0.07)	0.08 (0.07)
B_5_	−0.52 (0.05) ***	−0.52 (0.05) ***	−0.51 (0.05) ***	−0.52 (0.05) ***	−0.52 (0.05) ***	−0.52 (0.05) ***
B_6_	0.11 (0.04) **	0.10 (0.04) **	0.11 (0.04) **	0.10 (0.04) **	0.11 (0.04) **	0.10 (0.04) **
B_7_	0.14 (0.03) ***	0.14 (0.03) ***	0.14 (0.03) ***	0.14 (0.03) ***	0.15 (0.03) ***	0.14 (0.03) ***
B_8_	0.01 (0.04)	0.01 (0.04)	0.02 (0.04)	0.01 (0.04)	0.03 (0.04)	0.01 (0.04)
R^2^	0.210	0.214	0.201	0.210	0.186	0.202
*F* (df)	32.60 (6, 738) ***	33.71 (7, 737) ***	24.92 (6, 738) ***	22.33 (7, 737) ***	23.08 (6, 738) ***	21.51 (7, 737) ***
*F* vs. b (df)	3.94 (1, 736) *	–	6.03 (2, 736) **	3.50 (1, 736)	12.97 (2, 736) ***	10.79 (1, 736) **
AIC	−162.157	−164.268	−154.163	−160.730	−140.467	−153.428
BIC	−129.863	−127.361	−121.870	−123.823	−108.173	−116.521

Note: Model: Y _Z reading achievements_ = D_1_ × (B_0_ + B_1_ × (X _Z home supervision_ − C) + B_4_ × X _gender_ + B_5_ × X _age_ + B_6_ × X _Z parental education_ + B_7_ × X _Z parental occupation_ + B_8_ × X _Z monthly income_) + D_2_ × (B_0_ + B_3_ × (X _Z home supervision_ − C) + B_4_ × X _gender_ + B_5_ × X _age_ + B_6_ × X _Z parental education_ + B_7_ × X _Z parental occupation_ + B_8_ × X _Z monthly income_). D_1_ = *rs11629841* TT carriers; D_2_ = *rs11629841* GT carriers; CI = confidence interval. *F* vs. b represents the *F*-test between the other nested models and model b; ^a^ represents that the parameter is limited to the specified value, while C = 1.85 indicates that the cross-over point C is fixed at the maximum value of 1.85 for home supervision; C = −4.31 indicates that the cross-point C is fixed at the minimum value of −4.31 for home supervision; B_1_ = 0 indicates a non-risk/non-plastic allele/genotype with no predictive effect of home supervision on the reading achievements of children, which is one of the assumptions of the strong differential susceptibility model/diathesis–stress model/vantage sensitivity model. B_3_ represents the predictive effect of home supervision on the reading achievements of children with a risk/plastic allele/genotype. B_4_ represents the predictive effect of gender on the children’s reading achievements; B_5_ represents the predictive effect of age on the children’s reading achievements; B_6_ represents the predictive effect of parental education on the children’s reading achievements; B_7_ represents the predictive effect of parental occupation on the children’s reading achievements; and B_8_ represents the predictive effect of family monthly income on the children’s reading achievements. * *p* < 0.05, ** *p* < 0.01, *** *p* < 0.001.

## Data Availability

The data that support the findings of this study are available from the corresponding author, L.Z., upon reasonable request.
